# Supplementary material for: Dub3 inhibition suppresses breast cancer invasion and metastasis by promoting Snail1 degradation
Source: Nat Commun. 2017 Feb 15;8:14228. doi: 10.1038/ncomms14228 (PMC5316870; doi:10.1038/ncomms14228)

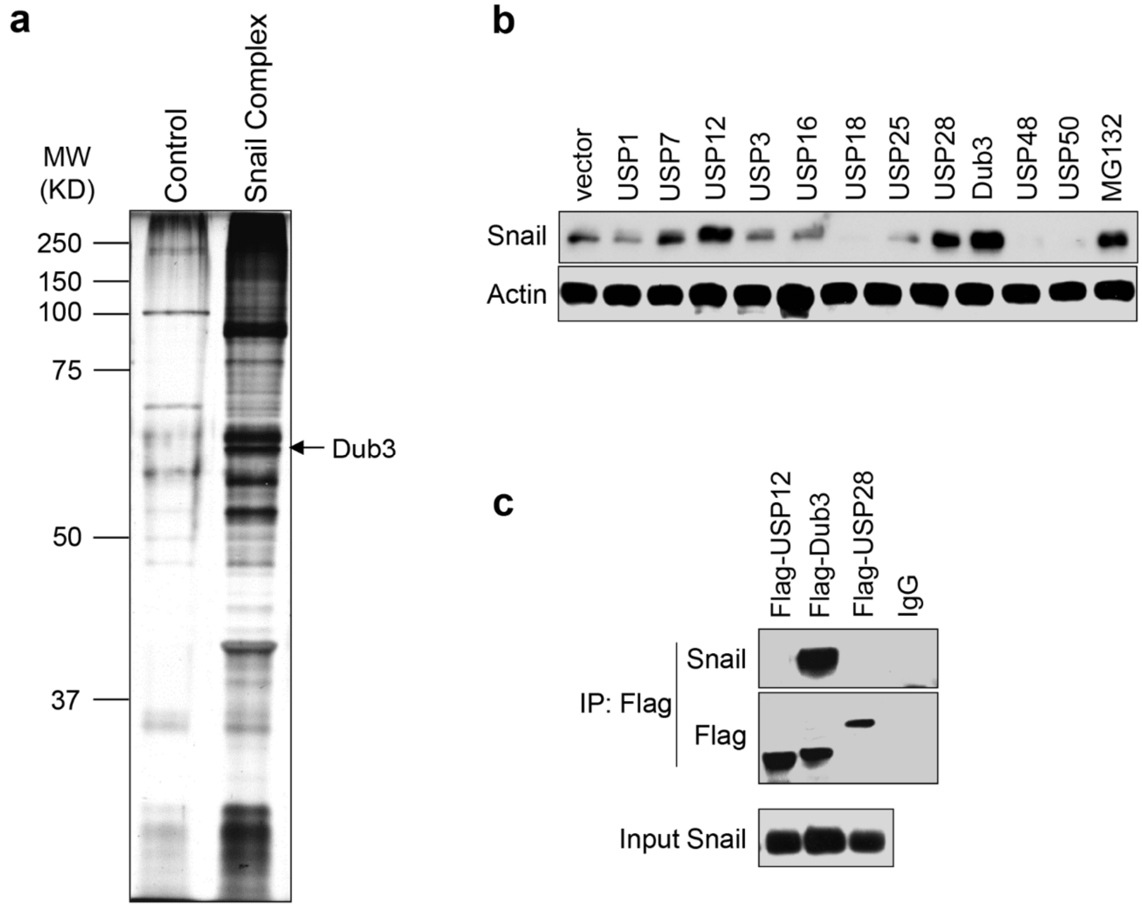

Supplementary Figure 1 | Dub3 stabilizes Snail1.

(a) The Snail1 complex was isolated by immunopurification, separated on SDS–PAGE and visualized by silver staining. A protein with molecular weight close to 65 kDa was excised and identified as Dub3 by mass spectrometry.

(b) Flag-Snail1 and indicated DUBs were co-expressed in HEK293 cells for 48 hr. Flag-Snail1 was also expressed in HEK293 cells for 42 hr followed by treatment with 10 μM MG132 for 6 hr. The protein level of Snail1 was analyzed by western blot with Flag antibody.

(c) HA-tagged Snail1 and Flag-tagged DUBs were co-expressed in HEK293 cells. After immunoprecipitation, bound Snail1 was examined by western blot.

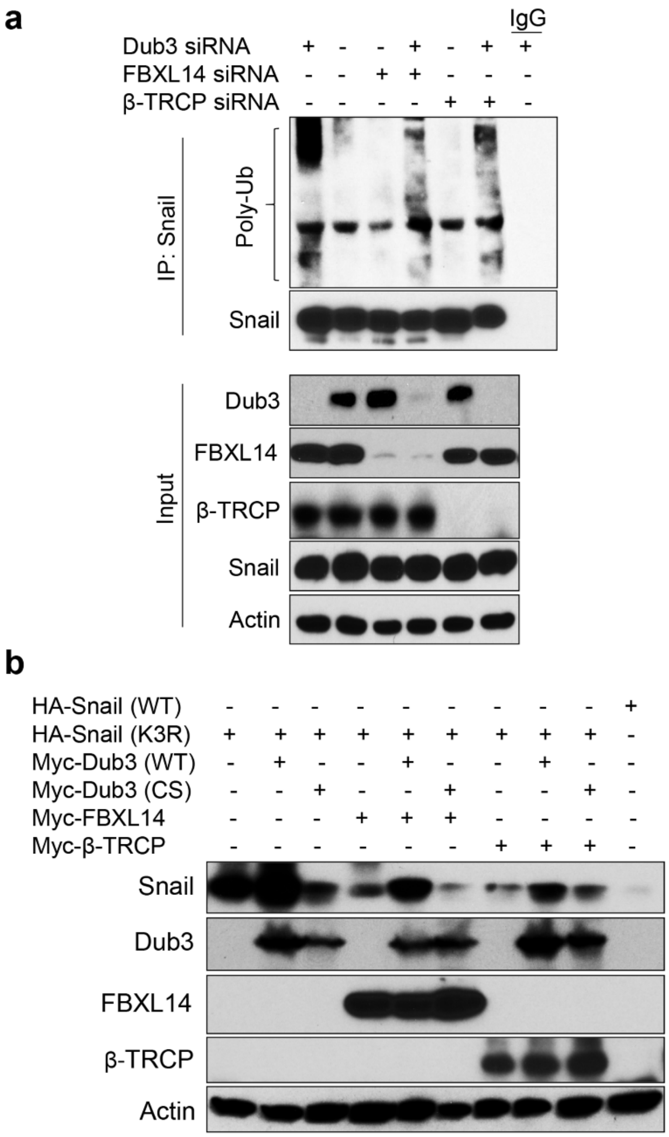

Supplementary Figure 2 | Dub3 deubiquitinates Snail1 and antagonizes the function of Snail1's E3 ligase.

(a) MDA-MB231 cells were transfected with siDub3, siβ-TRCP1 or SiFBXL14 alone or in combination. After 42 hr of post-transfection, cells were treated with MG132 for 6 hr and harvested for ubiquitination assessment.

(b) HEK293 cells were transfected with indicated set of expression plasmids, and cell lysates were analyzed for the expression of Snail1, Dub3, β-TRCP1 and FBXL14 by western blot.

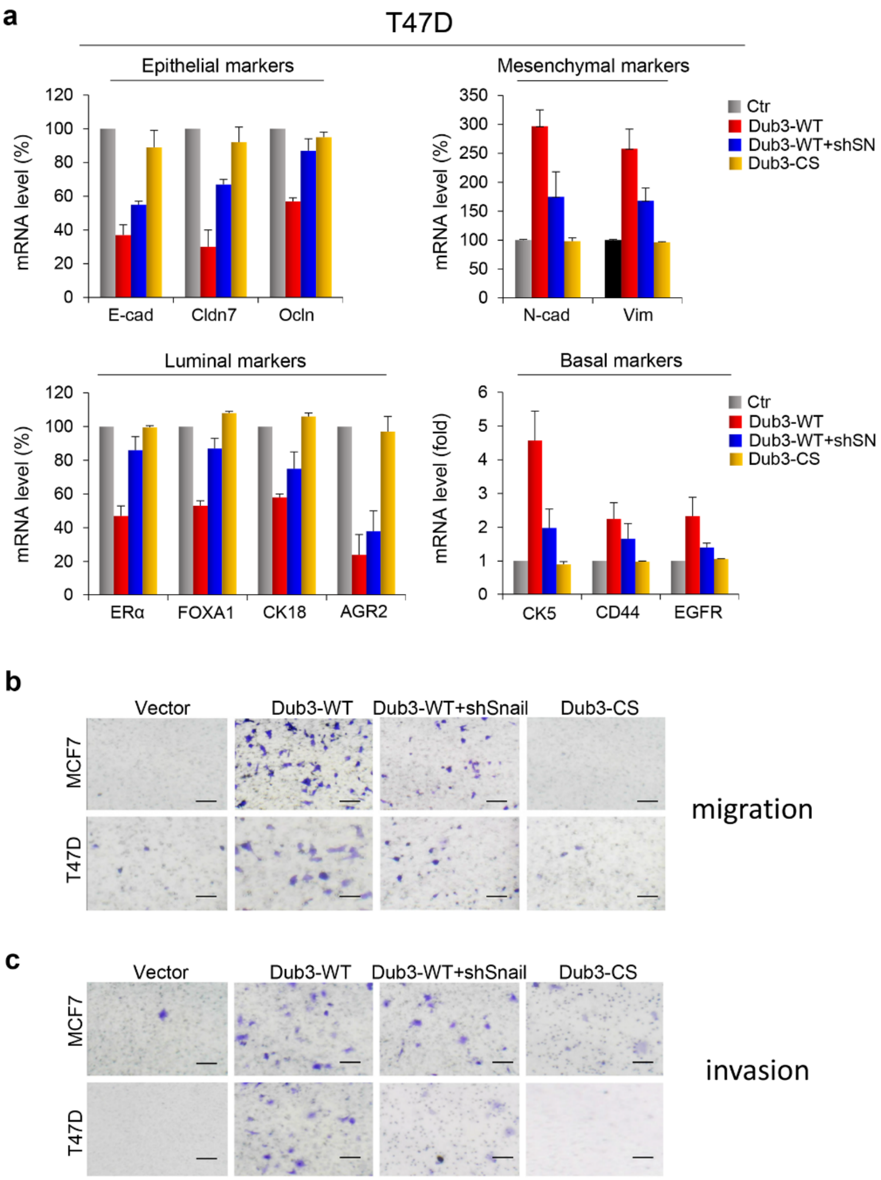

Supplementary Figure 3 | Overexpression of Dub3 induces EMT.

(a) WT- or CS-Dub3 was expressed in T47D cells. A rescue experiment with knockdown of Snail1 expression in WT-Dub3 expressing cells was also performed. The mRNA levels of epithelial, mesenchymal, luminal, and basal markers were quantitated by real-time PCR. Data are shown as mean ± SD of two separate experiments in triplicates.

(b) Representative images of Boyden chamber migration assay of modified MCF7 and T47D cells, as described in (a). Scale bar = 200 μm

(c) Representative images of Boyden chamber invasion assay of modified MCF7 and T47D cells, as described in (a). Scale bar = 200 μm

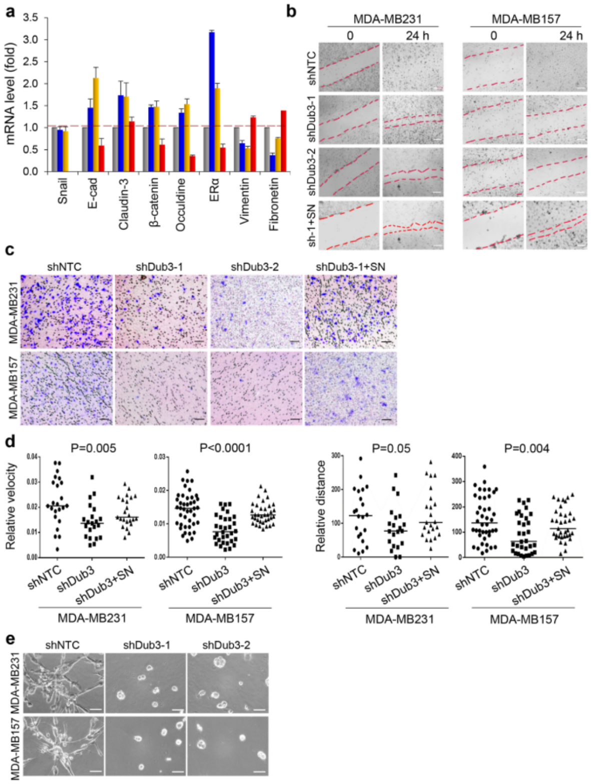

Supplementary Figure 4 | Knockdown of Dub3 inhibits migration, invasion in BLBC cells by downregulation of Snail1.

(a) Real-time PCR analysis of the mRNA level of several EMT markers in MDA-MB231 cells. Data are shown as mean ± SD of two separate experiments in triplicates.

(b) The migratory ability of MDA-MB231 and MDA-MB157 cells and the corresponding stable transfectants with knockdown of Dub3 expression or Snail1 rescued expression in Dub3 knockdown cells were analyzed by wound healing assay. A representative experiment is shown. Scale bar = 200 μm.

(c) The invasiveness of MDA-MB231 and MDA-MB157 cells stably expressed control vector, Dub3 shRNA or Snail1 rescued expression in Dub3-knockdown cells was analyzed with a modified Boyden chamber invasion assay. A representative experiment is shown. Scale bar = 200 μm.

(d) A total of 30 cells were scored for distance and velocity and shown graphically. Each point represents the track of one individual cell, and the bar displays the median value. ***P>0.005 as determined by one-way analysis of variance.

(e) 3D morphology of MDA-MB231 and MDA-MB157 cells expressed control vector or Dub3 shRNA. Scale bar = 500 μm.

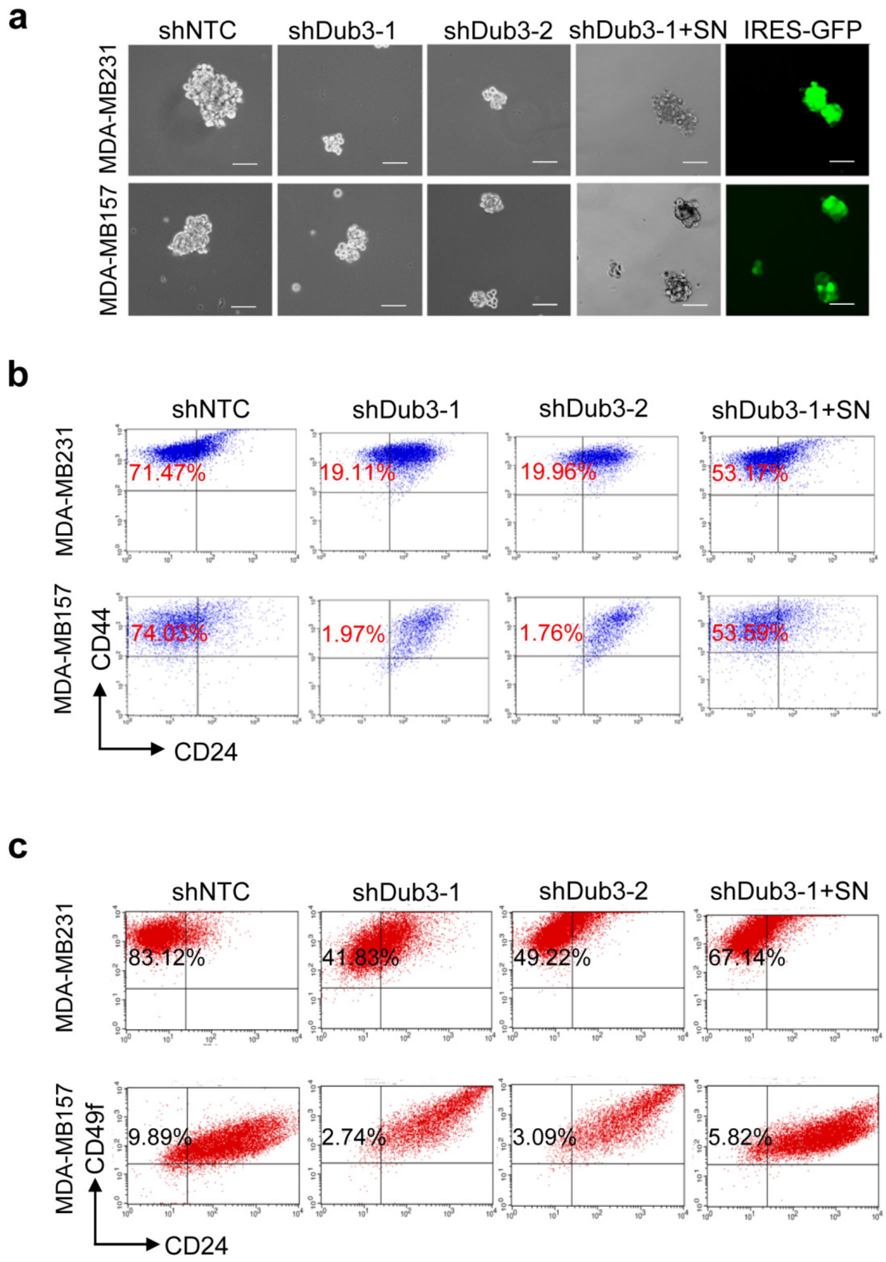

Supplementary Figure 5 | Knockdown of Dub3 inhibits CSC-like characteristics in BLBC cells by downregulation of Snail1.

(a) Representative images of tumorsphere-formation from MDA-MB231 and MDA-MB157 cells stably expressed control vector, Dub3 shRNA or Snail1 rescued expression in Dub3-knockdown cells. Scale bar = 200 µm.

(b) Representative images of the CD44^{high}/CD24^{low} population sorted from cells described in (a).

(c) Representative images of the CD49f^{high}/CD24^{low} population sorted from sorted from cells described in (a).

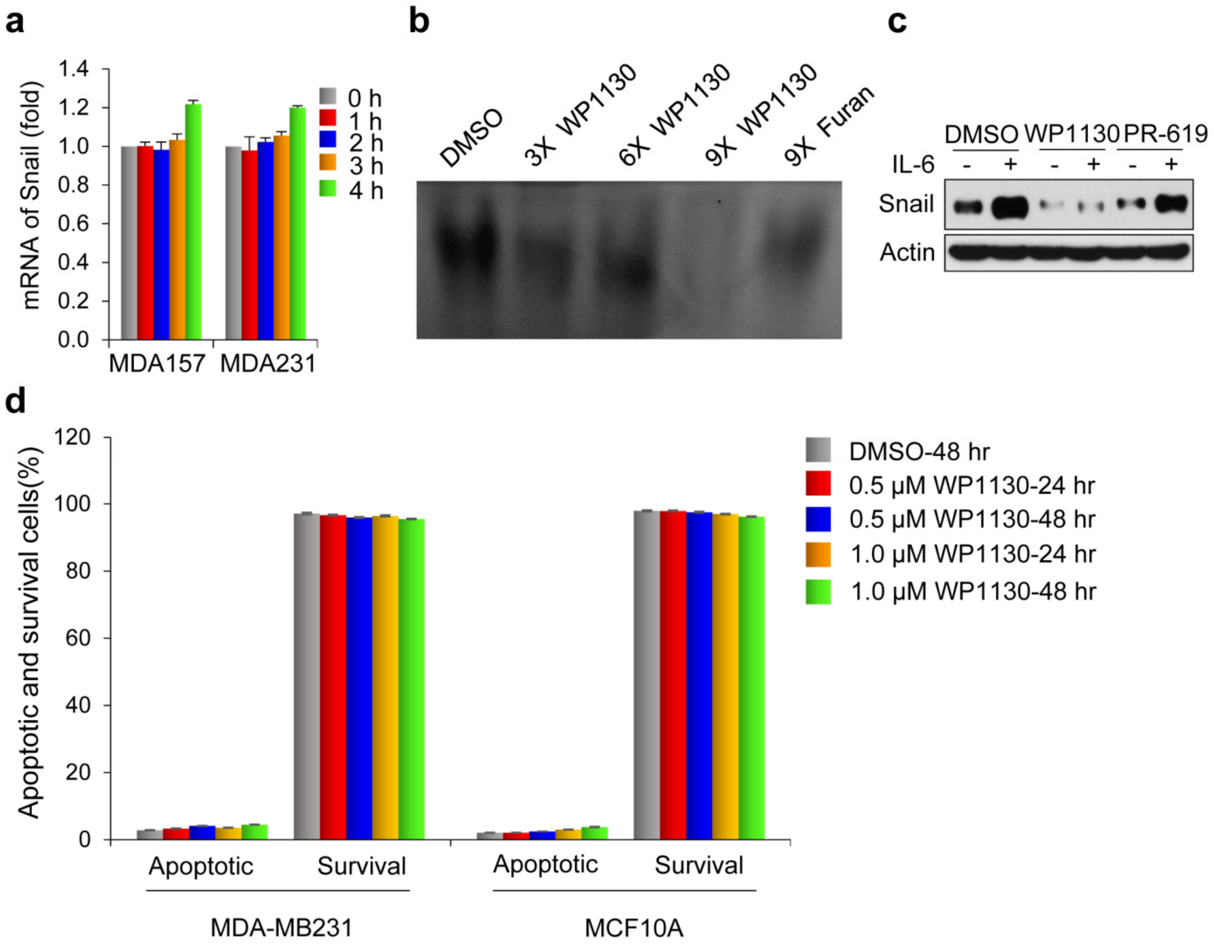

Supplementary Figure 6 | WP1130 is an inhibitor of Dub3.

(a) Real-time PCR analysis of Snail1 mRNA in MDA-MB231 and MDA-MB157 cells after IL-6 treatment with different time intervals. Data are shown as mean ± SD of two separate experiments in triplicates.

(b) Interaction of WP1130 with purified recombinant Dub3 shifted the apoenzyme of Dub3 (DMSO), whereas Furan did not have this effect. This interaction appears to be dose-dependent and cause protein destabilization or degradation (especially in lane 4).

(c) MDA-MB231 cells were serum starved for 24 hr and then treated with WP1130 or PR619 for half hour followed by IL-6 treatment for 2 hr. The cell lysate was analyzed with western blot.

(d) Immortalized normal human breast epithelial cells (MCF10A) and MDA-MB231 cells were treated with different doses of WP1130 (0.5 and 1 μM) for different time intervals (24 and 48 hrs). The apoptotic and survival cells were analyzed by FACS. Data are shown as mean ± SD of two separate experiments in triplicates.

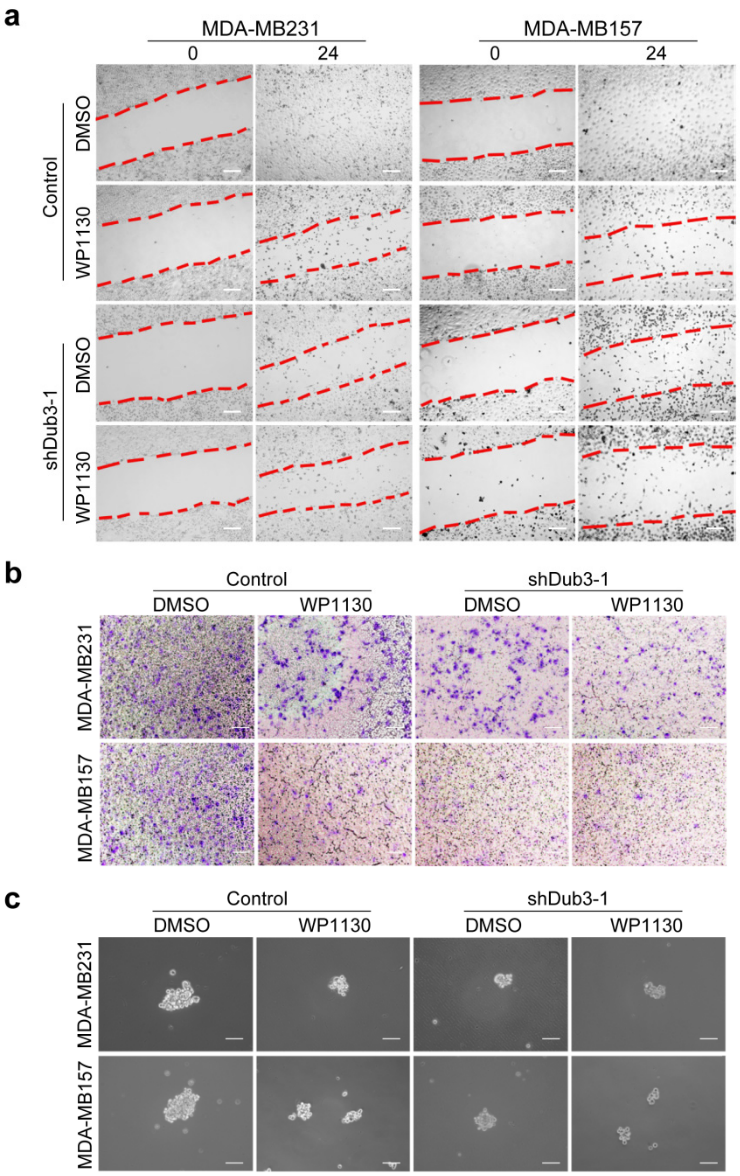

Supplementary Figure 7 | WP1130 inhibits Dub3 activity.

(a) MDA-MB231 and MDA-MB157 cells stably expressing control vector or Dub3 shRNA were treated with 0.5 μM WP1130 for 24 hr and analyzed for cell migration using wound healing assay. Representative images are shown. Scale bar = 200 μm.

(b) Cells as in (a) were treated with 0.5 μM WP1130 for 4 hr and analyzed for cell invasion. Representative images are shown. Scale bar = 200 μm.

(c) Cells as in (a) were treated with 0.5 μM WP1130 and analyzed for tumorsphere-formation. Representative images are shown. Scale bar = 200 μm.

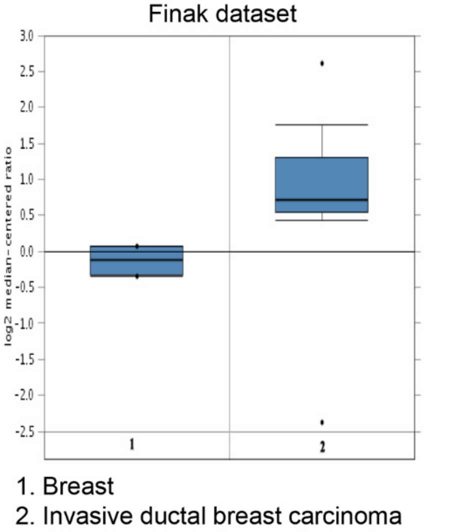

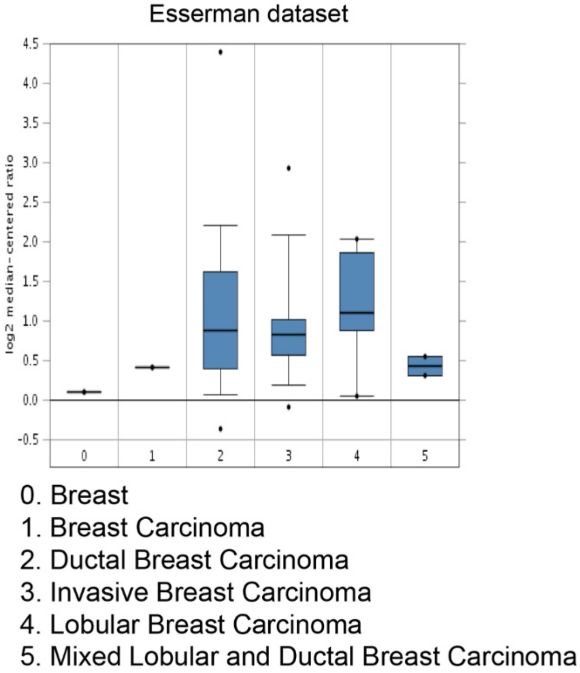

Supplementary Figure 8 | Expression of Dub3 and Snail1 are positively correlated in breast cancer patients.

Based on gene expression profiles (datasets from Finak and Esserman in Oncomine), high level of Dub3 mRNA was found in the invasive ductal breast carcinoma compared to normal breast tissues.

Supplementary Figure 9 | Full scans of the most important blots.

Figure 1a

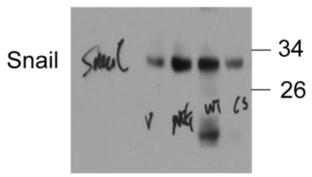

Figure 1b

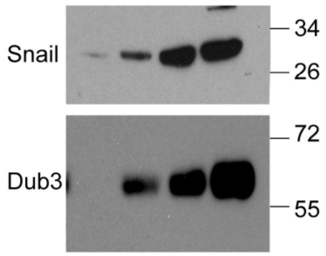

Figure 1d

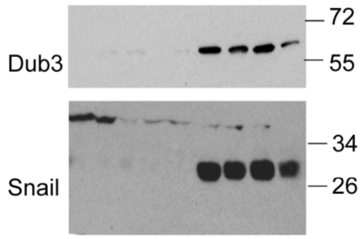

Figure 1g

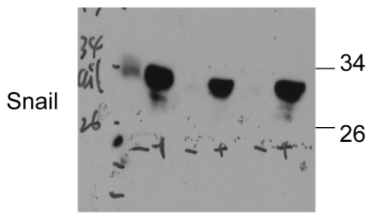

Figure 2e

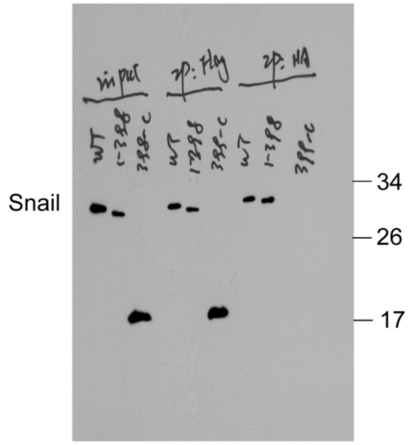

Figure 2g

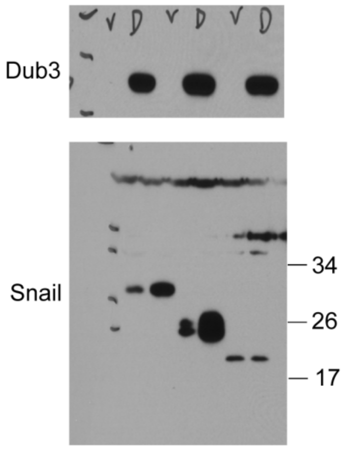

Figure 3a

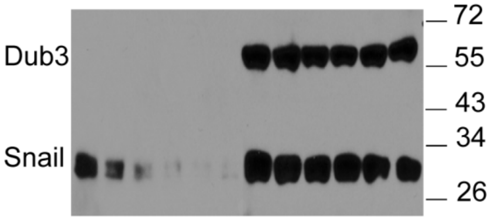

Figure 3d

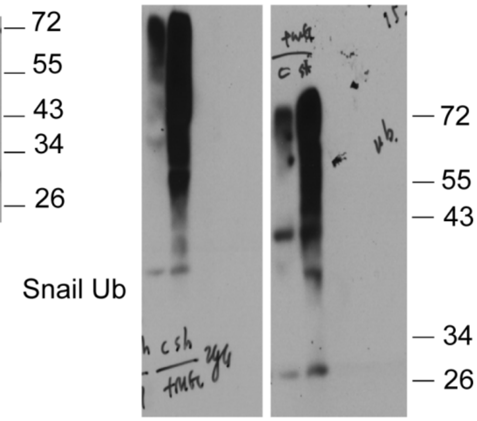

Figure 3g

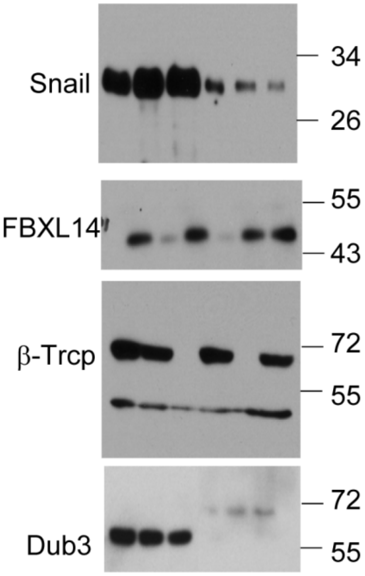

Figure 3b

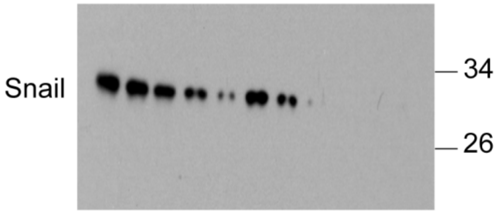

Figure 3e

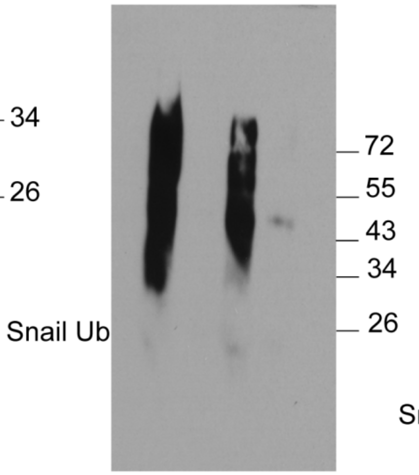

Figure 3h

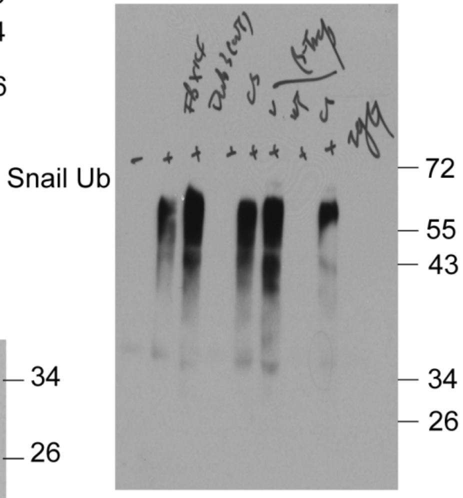

Figure 3c

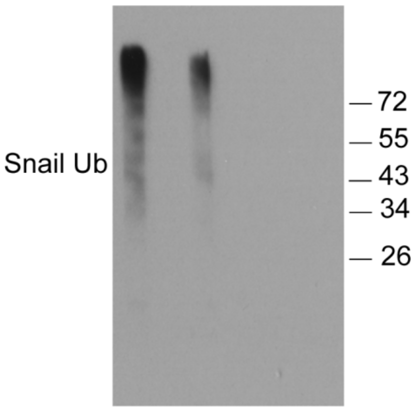

Figure 3f

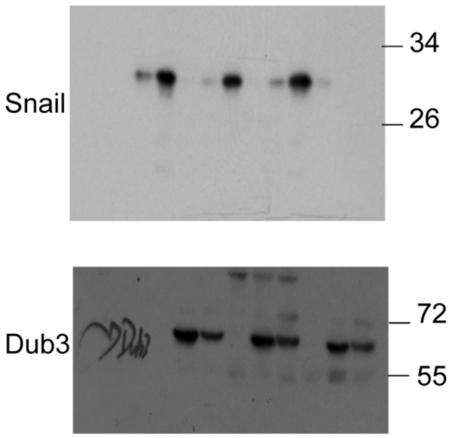

Figure 4a

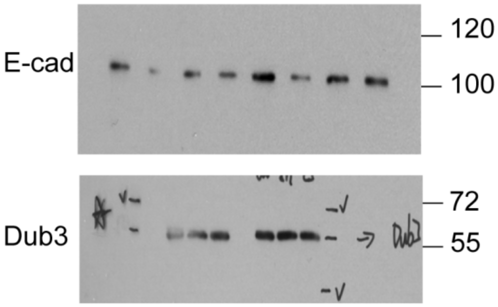

Figure 5a

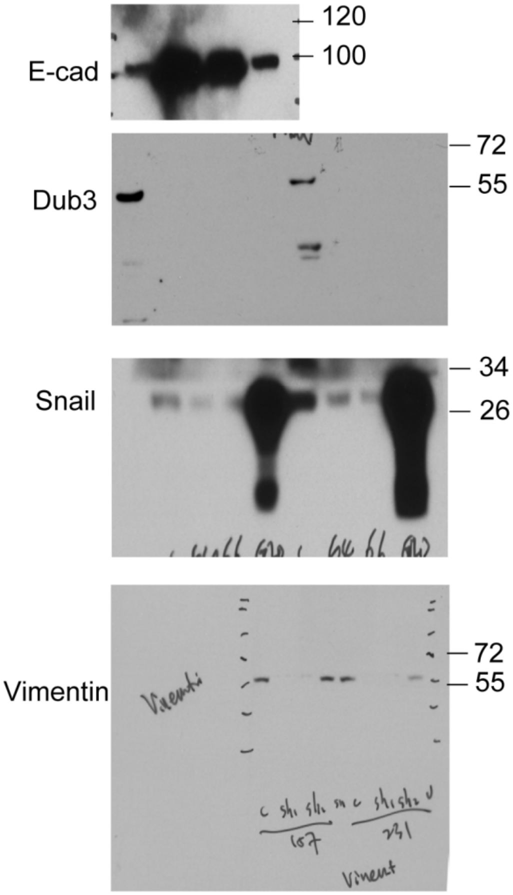

Figure 6e

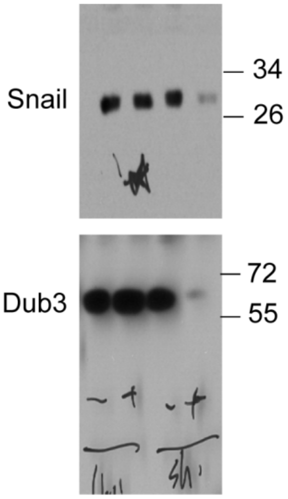

Figure 7a

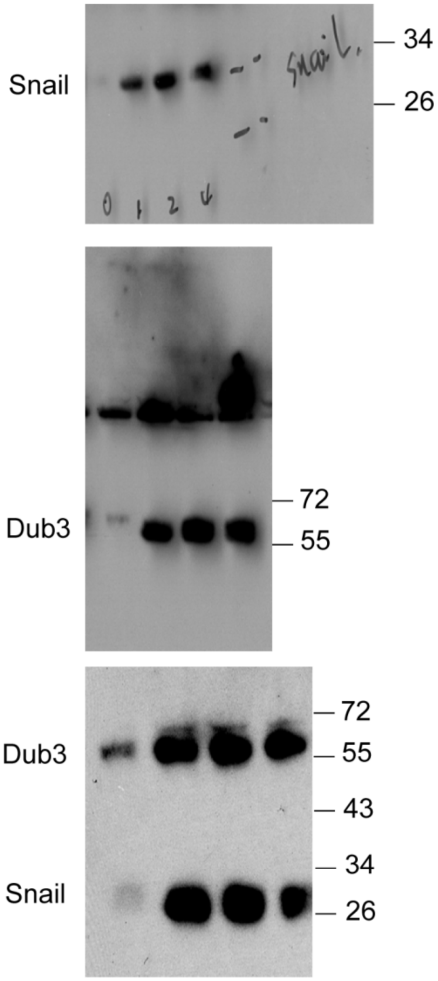

Figure 7b

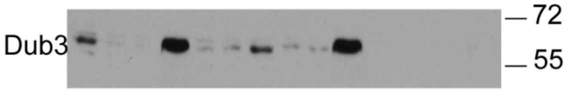

Supplement: Supplementary Information — Supplementary Figures [file ncomms14228-s1.pdf]
